# Temperament, Character, and Adolescents' Depressive Symptoms: Focusing on Affect

**DOI:** 10.1155/2012/925372

**Published:** 2012-07-15

**Authors:** Danilo Garcia, Nóra Kerekes, Ann-Christine Andersson Arntén, Trevor Archer

**Affiliations:** ^1^Institute of Neuroscience and Physiology, The Sahlgrenska Academy, University of Gothenburg, Wallinsgatan 8, 431 41 Gothenburg, Sweden; ^2^Centre for Ethics, Law, and Mental Health (CELAM), University of Gothenburg, Wallinsgatan 8, 431 41 Gothenburg, Sweden; ^3^R&D Unit, Swedish Prison and Probation Service, Wallinsgatan 8, 431 41 Gothenburg, Sweden; ^4^Department of Psychology, University of Gothenburg, Box 500, 405 30 Gothenburg, Sweden

## Abstract

Positive (PA) and negative affect (NA) are two separate systems markers of subjective well-being and measures of the state depression (low PA combined with high NA). The present study investigated differences in temperament, character, locus of control, and depressive symptoms (sleep quality, stress, and lack of energy) between affective profiles in an adolescent sample. Participants (*N* = 304) were categorized into four affective profiles: “self-fulfilling” (high PA, low NA), “high affective” (high PA, high NA), “low affective” (low PA, low NA), and “self-destructive” (low PA, high NA). Personality was measured by the Temperament and Character Inventory and affective profiles by the Positive Affect and Negative Affect Schedule. The “self-fulfilling” profile was characterized by, compared to the other affective profiles, higher levels of sleep quality, less stress and more energy and also higher levels of persistence and a mature character (i.e., high scores in self-directedness and cooperativeness). “Self-destructive” adolescents reported higher levels of external locus of control, high scores in harm avoidance and reward dependence combined with less mature character. The results identify the importance of character maturity in well-being and suggest that depressive state can be positively influenced by promoting positive emotions which appears to be achieved by character development.

## 1. Introduction

Although most would agree in viewing positive and negative affect as opposite ends of a continuum, there is much evidence that they are best construed as two separate systems [1]. For example, the two dimensions are also measures of clinical diagnoses, such as depression, defining it as a mixed state of high negative affect (NA) and low positive affect (PA) [2]. An increase in the frequency of PA and a decrease in the frequency of NA, or a combination of both changes, may lead to an improvement of the depressive symptoms [3]. This simple observation might be crucial regard in adolescence—a period of life when daily problems (e.g., coping with a minor social conflict) seem to be equally stressful experiences as major life events (e.g., parent being remarried) [4].

Moreover, the two dimensions of affect are markers of subjective well-being (SWB), which is usually measured through subjective evaluation and involves both a cognitive and an affective component [5]. Life satisfaction is suggested as the cognitive component and refers to a comparison process in which individuals assess the quality of their lives on the basis of their own self-imposed standard [6]. The affective component of SWB is computed by subtracting the number of NA from the number of PA experienced by the individual, resulting in the so-called affect balance [7]. Seeing the affective component as a unidimensional model, with “ill-being” at one pole and “well-being” at the other, present qualitative differences between individuals that are high compared to low in both dimensions [8].

 Along this line of reasoning, some researchers [9–11] have developed the affective personality profiles based on self-reported affect measured by the Positive Affect and Negative Affect Schedule [12]. The combinations of these two variables generate four different profiles: self-fulfilling (high PA, low NA); high affective (high PA, high NA); low affective (low PA, low NA); and self-destructive (low PA, high NA). The affective personalities framework goes beyond the view of affect as a two separate systems, taking into account the interaction of both dispositions as recommended by Ito and Cacioppo [8] and also observations of two-system theories suggesting that, when using dichotomous features, combinations must be ruled out [13].

Studies among adults show that self-fulfilling and high affective profiles, compared to low affective and self-destructive profiles, perform better during stressful situations (e.g., Norlander et al. [9], induced stress using the Stroop Color and Word Test by Stroop [14]) have a more active life (i.e., exercise more often and more intensively). Even in physiological measures differences can be detected, such as they have lower blood pressure [9, 10] (see also Kunst [15] who showed that the self-destructive and high affective profiles were strongly associated with increased posttraumatic stress disorder symptoms severity). Nevertheless, while low affective profiles have responded maladaptive to induced stress, compared to self-fulfilling and high affective individuals [9], they have reported less stress in their life as the self-fulfilling profiles [10]. In the context of adolescence, low affective ones seem to avoid stressful situations by neutralizing negative and positive experiences (see Garcia and Siddiqui research [16], for results using the Interpretation and Recognition for Words in a Short Story measure [17], which indicate that low affective adolescents rate negative words in a short story as neutral). Self-fulfilling adolescents, just as adults, report feeling more energetic and optimistic than the other three affective profiles [11].

Moreover, self-acceptance [18, 19], locus of control [20], and self-efficacy [21] are also good predictors of adolescents' affective experience. These constructs are, at least in part, good definitions of character (i.e., concepts of the self, goals, and values) [22]. Although the concept of character holds a major position in psychology, see for example [23, 24], most research on happiness (e.g., high PA and low NA) has focused on traits models of personality, for example, the Big Five Model of personality [25].

According to Cloninger's psychobiological model of personality [26], temperament reflects the basic organization of independently different brain systems for the activation, maintenance, and inhibition of behaviour in response to stimuli. Hence, temperament may be responsible for our likes and dislikes or what Haidt [27] calls the “Like-o-Meter”. Character is defined as what people make of them selves intentionally or individual differences in prepositional learning of personal goals, values, and even defence mechanisms [22]. Cloninger [22] has suggested that character modifies the significance or meaning of what is experienced, hence also changing emotional reactions and habits. In other words, understanding the self as a unity of being (the self as an autonomous individual, the self as an integral part of humanity or society, and the self as an integral part of the universe) leads to attitudes that increase “personal satisfaction, sublimation, and flexibility regardless of external circumstances” ([22, page 126]; see Table 1 for a closer description of the different dimensions of Cloninger's psychobiological model).

Cloninger's psychobiological model of personality offers important contributions to the understanding of the role of personality on adolescents' emotional experience. Adolescents who seek novel experiences (high novelty seeking) and higher levels of reward (high reward dependence), for example, often engage in risky behaviors, without considering future outcomes or consequences [28], thereby explaining why some extrovert behaviours might be counterproductive and lead to high levels of NA in the context of adolescence. Moreover, self-directedness, a character dimension defined by autonomous and goal-directed behaviour, self-control, and the sense of responsibility is negatively related to NA, while harm avoidance, a temperament dimension characterized by excessive worrying, pessimism, and being fearful, doubtful, and easily fatigued, is positively related to NA [29]. Development of the self in adolescence may be focused on the concept of the self as independent (i.e., self-directed behaviour) [30] and that specific concept of the self may be another key element to understand the experience of high PA—being able to withstand peer pressure, for instance, gives feelings of personal integrity, honour, self-esteem, effectiveness, leadership, and hope. Such character strengths and feelings may lead to positive affect because they strengthen and prepare the adolescent to cope with different situations [31–34]. More recently, neuroimaging research suggests that cognitive and behavioral changes occurring during adolescence might be understood from the perspective of increased “executive functioning” (e.g., attention, response inhibition, regulation of emotion, organization, and long-range planning; for a review see [35]). This high orders functioning relies on frontal lobe circuitry and represents character maturing.

Accepting that PA and NA are different constructs, the present study investigates differences in temperament, character, locus of control, and symptoms of depression (sleep quality, stress, and lack of energy) between affective personality profiles in an adolescent sample. The hypothesis that self-destructive adolescents report most depressive symptoms compared to their classmates, and that personality profiles (including temperament and character) are distinct descriptive of affective profiles is tested.

## 2. Method

### 2.1. Participants

The 304 participants (183 boys, 121 girls) were high school pupils from west Sweden (*M = *17.34 years, SD = 1.16, range = 16–19). They were from different socioeconomic and cultural backgrounds and specializing in different subjects during their studies.

### 2.2. Instruments

**Positive Affect and Negative Affect Schedule (PANAS) [12]**

The Swedish PANAS version used in the present study has been largely used in other studies among adolescents, for example, [36–39]. The PANAS instructs participants to rate to what extent they generally have experienced 20 different feelings or emotions (10 PA and 10 NA) for the last four weeks, using a 5-point Likert scale (1 = very slightly, 5 = extremely). The 10-item PA scale includes adjectives such as strong, proud, and interested. The 10-item NA scale includes adjectives such as afraid, ashamed, and nervous. In the present study, PA showed a Cronbach's *α* = .83 and NA a Cronbach's *α* =  .83.

Previous studies [9–11] have modified and developed the PANAS instrument further through a subject-response based derivation of the four types of affective personality profiles. This procedure was implemented in the present study through dividing the results on the PA scale into two parts thereby distributing the participants into one group with high PA and another group with low PA (cut-off point= 53.2%). The same procedure was implemented for the participants' responses on the NA-scale (cut-off point= 48.9%). Following this, the results from these two scales were combined according to the procedure that assigned each one of the participants into one of the four affective personality groups as follows: individuals showing high PA and low NA (self-fulfilling), high PA and high NA (high affective), low PA and low NA (low affective), and low PA and high NA (self-destructive).

**The Temperament and Character Inventory (TCI) [26]**

The TCI measures the seven factors of the psychobiological model of personality with a total of 238 items with forced binary answer (yes or no). The Swedish version of the TCI [40] was used in the present study. The four temperament dimensions are: harm avoidance (e.g., “I often feel tense and worried in unfamiliar situations, even when others feel there is little to worry about”), novelty seeking (e.g., “I often try new things just for fun or thrills, even if most people think it is a waste of time”), reward dependence (e.g., “I like to discuss my experience and feelings openly with friends instead of keeping them to myself”), and persistence (e.g., “I often push myself to the point of exhaustion or try to do more than I really can”). The three character dimensions are: self-directedness (e.g., “In most situations my natural responses are based on good habits that I have developed”), cooperativeness (e.g., “I often consider another person's feelings as much as my own”), and self-transcendence (e.g., “I sometimes feel so connected to nature that everything seems to be part of one living organism”). Cronbach's *α* varied between .69 and .72 in the present study.

**Stress and Energy (SE) [41]**

The SE instrument is a self-estimation scale, consisting of 12 items that assess individuals' experience of their own stress and energy. The test is divided into two subscales that express each participant's level of mood in the two dimensions: “experienced stress” and “experienced energy”. Response alternatives are ordered within 6 points Likert scales (0 = not at all, 5 = very much). The Cronbach's *α* were .64 for stress and .66 for energy in the present study.

**Uppsala Sleep Inventory (USI) [42]**

The USI is a self-report instrument describing participants' sleep profiles, as characterized by descriptions of potential difficulties falling asleep, psychophysiological problems (such as body aches, muscle tension, beating heart, “pins and needles”, anxiety feelings, etc.), and larger sleep problems. Here five response alternatives were available: “*none*”, “*a little*”, “*about average*”, “*large*”, and “*very great*”. The Cronbach's *α* were .71 for major sleep problems, .75 for difficulties falling asleep, and .72 for psychophysiological problems.

**Locus of Control [43]**

The Locus of Control scale is a modified version of the original Rotter scale [44]. The scale has a minimum score of 8 and a maximum of 40, with a lower score representing an external locus of control orientation and a higher score representing an internal locus of control orientation.

### 2.3. Procedure

All adolescents in the study had written parental consent. If parents wanted any further information about the study, they were asked to contact the researchers. All adolescents received cinema tickets for their anonymous participation.

For statistical analyses the multiple analysis of variance (MANOVA) test was applied in order to identify differences in depressive symptoms and personality between affective profiles, during what the affective profile was the independent variable and the dependent variables were depressive symptoms (i.e., levels of stress and energy, sleep problems, difficulties falling asleep, psychophysiological problems), personality (measured by the TCI), and locus of control (external and internal).

## 3. Results

The four defined affective profiles were characterized with different patterns of depressive symptoms. A significant effect emerged for levels of stress (*F*(3, 321) = 19.03, *P* < .001), energy (*F*(3, 321) = 20.31, *P* < .001), large sleep problems (*F*(3, 321) = 18.01, *P* < .001), difficulties falling asleep (*F*(3, 318) = 24.45, *P* < .001), and for psychobiological problems (*F*(3, 321) = 11.19, *P* < .001). Self-fulfilling adolescents reported significantly higher level of energy as well as in all cases lower (in most cases significantly lower) levels of stress, sleep, or psychobiological problems (Table 2). The group of high affective ones differed mostly from self-destructive ones in their increased energy. The low affective adolescents differed mostly from the self-destructive ones in their decreased level of stress and psychobiological problems.

In regard to personality profiles describing the four affective group self-fulfilling adolescents were characterized with significantly lower harm avoidance (*F*(3, 303) = 23.30, *P* < .001), and significantly higher persistence (*F*(3, 303) = 7.63, *P* < .001), than the other three groups of youngsters. Moreover, they possessed a more mature character, measured with significantly higher scores in TCI self-directedness (*F*(3, 303) = 20.55, *P* < .001) and TCI cooperativeness (*F*(3, 303) = 2.59, *P* = .05). On the other side, high affective and self-destructive groups were described by high reward dependence. Low-affective adolescents differed mostly in lower self-transcendence (*F*(3, 303) = 6.61, *P* < .001) from their high affective classmates. Adolescents with a self-destructive profile reported higher external locus of control (*F*(3, 317) = 9.07, *P* < .001) while no differences emerged for internal locus of control (*F*(3, 320) = 1.30, *P* = .263) between any of the four groups (Table 3).

## 4. Discussion

In the present study, we tested the hypothesis that self-destructive adolescents report most depressive symptoms compared to their classmates, and that personality profiles (including temperament and character) are distinct descriptive of affective profiles. The results showed that adolescents expressing a self-fulfilling profile (i.e., high PA and low NA) report higher levels of sleep quality, less stress, and more energy than the other three profiles and had a more mature character and were more persistent. As suggested in the introduction, adolescents who seek novel experiences and higher levels of reward often engage in risky behaviours, without considering future outcomes or consequences [28]. In contrast, character strengths may lead to positive emotions because they strengthen and prepare the adolescent to cope with different situations [22, 31]. Although Cloninger [22] describes possible risks throughout life for persons with special combinations of “difficult temperament” mediated by psychosocial conflicts, the temperament is assumed to have a more pathoplastic effect while the character development is the key to understand and evaluate health. Recently, Brown and colleagues [45] found that, among adolescents, PA was positively correlated with mindfulness measured with Mindful Attention Awareness Scale Adolescent. Mindfulness, in turn, was positively related to higher life satisfaction and wellness. In day-to-day life, mindfulness is an awareness of the presence and a conscious processing based on experience [46]. Positive affect has also been found to be related to and associated with better stress handling [9, 47], better sleep quality [48], and better coping resources [10, 47]. Even momentarily PA can be associated with such positive effects as survival in elderly people [49]. Together, these findings indicate that PA is associated with a more “mature” and health related character in adolescence.

In the present study, this mature character was defined by a higher autonomous self-concept (i.e., high in self-directedness) and a tendency to be more socially adapted (i.e., high cooperativeness). A self-directed adolescent might be described as mature and strong, responsible and reliable, purposeful, resourceful and effective, and self-accepted [22]. Similarly, an adolescent expressing high level of cooperativeness is socially tolerant, empathic, helpful, compassionate, and constructive [22]. Thus, interventions that promote such types of behaviours and attitudes to the self should be encouraged at home and in schools. Brown et al. [45] and Teasdale [46] suggests that interventions influencing adolescents to give and receive help (e.g., peer counselling) strengthens self-acceptance and fosters adolescents' capacity to experience moments of happiness and identity formation. The benefits from such interventions are greater “self-esteem, sense of purpose and worth, feeling of accomplishment and mastery, and satisfying interaction with other humans beings [sic]” [50, page 192]. These benefits are, at least in part, a good definition of self-directedness. With respect to self-transcendence, Magen's research suggests that adolescents address simple forms of this self-concept. Moreover, adolescents' pursuit of positive emotions tend to be egocentric and directed by their own desires [51]. Thus, these tendencies may be contradictory to the willingness to become dedicated to the well-being of others or prosocial causes that transcend the self [51]. Nevertheless, our findings show that high scores in the self-transcendence dimension might be related to high affectivity during adolescence.

Locus of control may be viewed as a prevailing expectancy, perceptions/cognitions that allow the evaluation of situations and circumstances. Individuals characterised by an internal locus of control (internals) believe that they control their own fate whereas those characterised by external locus of control (externals) are convinced that chance, luck, the stars, or behaviour of others determine the course of their lives. In the present study, individuals expressing self-fulfilling behaviour expressed markedly less (less than half) external locus of control than individuals expressing self-destructive. This observation associates high levels of external locus of control with depressive tendencies. Previously, in three different studies, Archer and colleagues [52] showed that external locus of control was predicted by NA and counter-predicted by PA and optimism. Higher scores of NA have also been found to be associated with a more externally based locus of control and a lower overall level of self-esteem among adolescents [53]. Marklund [54] obtained a strong positive correlation between “externals” and urgency (a factor involving impulsiveness) which implies that these individuals showed “difficulties resisting desires” and “temptations” together with the tendency to resort to impulsive behaviours in order to alleviate negative emotions; these observations imply also that an “external” strategy in viewing events relates notably to temperament. Generally, “externals” express lower levels of satisfaction and rehabilitation and lesser health benefits compared to “internals” [52].

### 4.1. Limitations and Further Questions

Although the instruments used to measure PA and NA showed high reliability, appropriate measures for the two constructs have been developed and validated for use with adolescents (e.g., the PANAS-C) [55]. Nevertheless, evidence of the reliability and validity of the PANAS in adolescents can be found elsewhere [56]. It is also important to mention that there is a version for measuring the seven factors of Cloninger's personality model among youth: the Junior Temperament and Character Inventory (JTCI). However, the JTCI was developed for use with children between the ages of 9 and 13 [57]. The version of the TCI used in the present study was found more appropriate because the participants in the present study were high-school pupils with an age range from 16 to 19.

Studies among preadolescent children may supply important insights of adolescents' depressiveness and related symptoms since predictors of behavioural problems that persist from adolescence to adulthood (e.g., conduct problems) are best assessed prior to adolescence [58]. Neurodevelopmental aspects of affective disorders draw attention to adverse environmental influences during early and late childhood that need to be addressed [59].

## 5. Conclusions

The results presented here show an expected and well matching nomological network between the measures indicating a chance to influence a depressive state in a positive way by promoting positive emotions as well as encourage character maturation. With an investment in increasing character maturity in adolescents, an increase in life energy and decrease in less sleep and psychosocial problems could be achieved. These results together with other evidences from studies applying PANAS and TCI [60–62] suggest that the affective personality profiles provide clues regarding the emotional, cognitive, motivational, and somatic categories of well-being states.

*“Temperament lies behind mood; behind will, lies the fate of character. Then behind both, the influence of family the tyranny of culture; and finally the power of climate and environment; and we are free, only to the extent we rise above these.” *John Burroughs **

## Figures and Tables

**Table 1:** Temperament and character description (reproduced with permission of C. R. Cloninger).

Temperament and character descriptors		
*Temperament*	*High scores*	*Low scores*
Harm avoidance	Worrying and pessimistic;	Relaxed and optimistic;
	fearful and doubtful;	bold and confident;
	shy;	outgoing;
	fatigable.	vigorous.
Novelty seeking	Exploratory and curious;	Indifferent;
	impulsive;	reflective;
	extravagant and enthusiastic;	frugal and detached;
	disorderly.	orderly and regimented.
Reward dependance	Sentimental and warm;	Practical and cold;
	dedicated and attached;	withdraw and detached;
	dependent.	independent.
Persistence	Industrious and diligent;	Inactive and indolent;
	hard working;	gives up easily;
	ambitious and overachiever;	modest and underachiever;
	perseverant and perfectionist.	quitting and pragmatist.
*Character*		
Self-directedness	Mature and strong;	Immature and fragile;
	responsible and reliable;	blaming and unreliable;
	purposeful;	purposeless;
	resourceful and effective;	inert and ineffective;
	self-accepted;	self-striving;
	habits congruent with long-term goals.	habits incongruent with long-term goals.
Cooperativeness	Socially tolerant;	Socially intolerant;
	empathic;	critical;
	helpful;	unhelpful;
	compassionate and constructive;	revengeful and destructive;
	ethical and principled.	opportunistic.
Self-transcendence	Wise and patient;	Impatient;
	creative and self-forgetful;	unimaginative and self-conscious;
	united with the universe.	pride and lack of humility.

**Table 2:** Mean (±SD) scores for stress, energy, large sleep problems, difficulties falling asleep, and psychobiological problems among affective profiles.

	Self-fulfilling	High affective	Low affective	Self-destructive
	(high PA and low NA)	(high PA and high NA)	(low PA and low NA)	(low PA and high NA)
Energy	30.49 ± .86*	26.02 ± .89	23.90 ± .88	21.70 ± .73
Stress	15.94 ± .99	20.16 ± 1.13^▲^	16.04 ± .86	26.50 ± 1.15^▲^
Major sleep problems	15.50 ± 4.20	19.30 ± 4.19^▲^	17.30 ± 4.69^▲^	19.95 ± 4.86^▲^
Difficulties falling asleep	17.42 ± 4.29	21.34 ± 4.96^▲^	18.52 ± 4.88	23.52 ± 6.49^▲^
Psychophysiological problems	20.61 ± .93	25.63 ± 1.07^▲^	24.06 ± 1.09^▲^	29.24 ± 1.09^▲^

*N* = 304, ^∗^higher than the other three groups, ^▲^higher than self-fulfilling.

**Table 3:** Mean (±SD) scores for temperament, character, and locus of control among affective profiles.

	Self-fulfilling	High affective	Low affective	Self-destructive
	(high PA and low NA)	(high PA and high NA)	(low PA and low NA)	(low PA and high NA)
Novelty seeking	56.06 ± 1.45	56.34 ± 1.58	55.37 ± 1.51	54.31 ± 1.53
Harm avoidance	33.22 ± 1.79	42.92 ± 1.35^▲^	45.19 ± 1.80^▲^	54.97 ± 1.93^▲^
Reward dependence	53.31 ± 1.48	56.58 ± 1.61^▲^	52.17 ± 1.55	57.06 ± 1.72^▲^
Persistence	59.38 ± 2.52^∗^	53.31 ± 2.72	43.50 ± 2.47	44.11 ± 2.41
Self-directedness	65.62 ± 1.45^∗^	58.02 ± 1.27	60.43 ± 1.73	48.30 ± 1.57
Cooperativeness	69.37 ± 1.39^∗^	67.13 ± 1.54	65.95 ± 1.59	63.05 ± 1.68
Self-transcendence	39.42 ± 1.87	48.61 ± 1.78^●^	34.77 ± 1.61	41.76 ± 1.73
External locus of control	7.11 ± 2.15	9.21 ± 2.33	7.25 ± 2.56	14.49 ± 2.32^▲^
Internal locus of control	12.71 ± 2.37	12.65 ± 2.88	12.41 ± 2.59	12.02 ± 2.46

*N* = 304, ^∗^higher than the other three groups, ^▲^higher than self-fulfilling, ^●^higher than low affective.
